# Effect of Cry1Ab Protein on Rhizobacterial Communities of Bt-Maize over a Four-Year Cultivation Period

**DOI:** 10.1371/journal.pone.0035481

**Published:** 2012-04-30

**Authors:** Jorge Barriuso, José R. Valverde, Rafael P. Mellado

**Affiliations:** Centro Nacional de Biotecnología (CSIC), Campus de la Universidad Autónoma, Cantoblanco, Madrid, Spain; Auburn University, United States of America

## Abstract

**Background:**

Bt-maize is a transgenic variety of maize expressing the Cry toxin from *Bacillus turingiensis*. The potential accumulation of the relative effect of the transgenic modification and the cry toxin on the rhizobacterial communities of Bt-maize has been monitored over a period of four years.

**Methodology/Principal Findings:**

The accumulative effects of the cultivation of this transgenic plant have been monitored by means of high throughput DNA pyrosequencing of the bacterial DNA coding for the 16S rRNA hypervariable V6 region from rhizobacterial communities. The obtained sequences were subjected to taxonomic, phylogenetic and taxonomic-independent diversity studies. The results obtained were consistent, indicating that variations detected in the rhizobacterial community structure were possibly due to climatic factors rather than to the presence of the Bt-gene. No variations were observed in the diversity estimates between non-Bt and Bt-maize.

**Conclusions/Significance:**

The cultivation of Bt-maize during the four-year period did not change the maize rhizobacterial communities when compared to those of the non-Bt maize. This is the first study to be conducted with Bt-maize during such a long cultivation period and the first evaluation of rhizobacterial communities to be performed in this transgenic plant using Next Generation Sequencing.

## Introduction

Rhizobacterial communities are a key element of soil quality and fertility [1]. The cultivation of genetically modified plants can alter these soil bacterial communities, hence endangering cultivar sustainability. Among these genetically modified maize, the so-called Bt-maize, harbours the *Bacillus thuringiensis* gene, which encodes the Cry protein, rendering the plant resistant to the attack of corn borer Lepidopterae.

The presence of the Bt protein potentially modifies the composition of root exudates of the transgenic plants and additionally may exerts a direct effect on non-target species of soil microorganisms [2], [3], [4], [5].

Many techniques have been used to analyse soil bacterial communities, including classic approaches based on the cultivation of viable bacteria, metabolic profiling studies and nucleic acid-based methods [6]. Most of the existing studies to date suggest that the cultivation of transgenic Bt-maize plants has minor effects or no effects at all on the rhizobacterial communities. However, these studies have not employed the Next Generation Sequencing (NGS) technique, which, upon being used for the sequencing of the SSU rRNA hypervariable regions, has proven to be very useful for the diversity studies of bacterial communities in many habitats, including soil [7], [8], [9], [10], [11]. Moreover, few studies are available for long-term periods of Bt-maize cultivation. We monitored the potential accumulation of the relative effects of Bt-maize on the rhizobacterial communities when compared to non-Bt maize by using NGS over a four-year period of cultivation. To this end, we have examined the structure of the rhizobacterial communities using a taxonomic approach [12], [13] conducted phylogenetic distances analyses [14], [15], [16], and bacterial diversity estimations. It has recently been reported that differences in diversity estimation may depend on the method used [17], [18], [19], therefore, we employed two commonly used methods to calculate diversity [12], [19], which have proven to be appropriate for the number and length of the sequences obtained [20].

To our knowledge, this is the first study conducted on rhizobacterial communities of Bt-maize during a four-year period using NGS, and the results obtained may be relevant as to a potential renewal of the authorisation to cultivate Bt-maize in the European Union.

## Results

**Figure 1:**
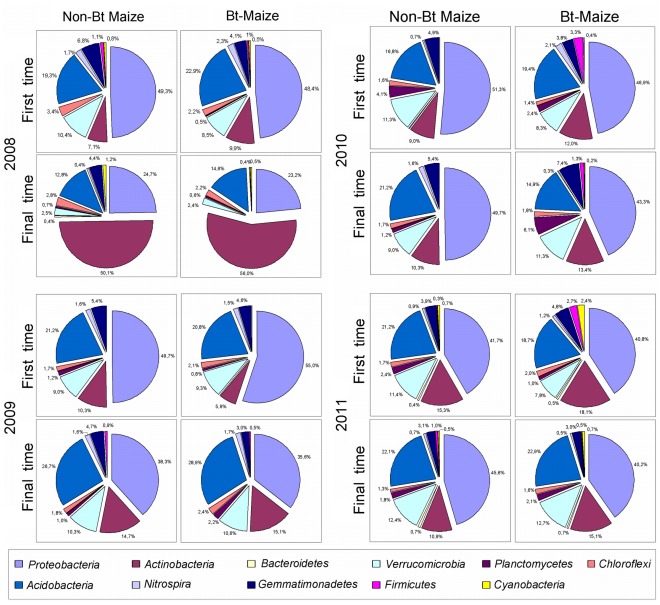
Taxonomic breakdown of the more relevant phyla. The percentages of *Proteobacteria*, *Actinobacteria, Bacteroidetes, Verrucomicrobia, Planctomycetes, Chloroflexi, Acidobacteria, Nitrospira, Gemmatimonadetes, Firmicutes* and *Cyanobacteria* are indicated and do not include the unassigned sequences. Unclassified sequences were not included as they were of no taxonomic use.

Sequences obtained from the Bt-maize and its non-transgenic counterpart at two different sampling times were compared with the sequences present in the RDP database and grouped using the MEGAN program and NCBI taxonomy to generate the taxonomic trees. Figure 1 shows the taxonomic breakdown as a result of sequencing the hypervariable V6 region of the 16S rDNA genes, and the corresponding taxonomic trees are included in figure S1 as Supplementary Information. The prominent phyla in all the samples were *Proteobacteria*, *Actinobacteria* and *Acidobacteria*.

A total of 1959 sequences were analysed from the rhizosphere of non-Bt maize at the first sampling time in the first year; 22.6% of these remained unassigned. From the assigned sequences, 49.3% belonged to the *Proteobacteria* phylum, 19.3% were *Acidobacteria*, 7.1% *Actinobacteria* and 23.7% belonged to other taxa. A very similar distribution of taxa (48.4% *Proteobacteria*, 22.9% *Acidobacteria,* 9.9% *Actinobacteria*, and 18.3% other taxa) was found for the 2445 total sequences from the Bt-maize rhizosphere at an equivalent sampling time, where 24.4% of sequences remained unassigned. At the final sampling time, *Proteobacteria* and *Acidobacteria* were again prominent among the assigned sequences: 24.7% and 12.8% respectively in the non-Bt maize (2680 total sequences), and 23.2% and 14.6% respectively in the Bt maize (2534 total sequences), while the presence of *Actinobacteria* markedly increased to 50.1% in the non-Bt maize and 56.0% in Bt maize. 23.6% and 21.2% were unassigned sequences respectively in the non-Bt and Bt-maize.

In the second year, the distribution of taxa in the assigned sequences of the non-Bt and Bt-maize was similar to that of the previous year at the first sampling time; *Proteobacteria*, *Actinobacteria* and *Acidobacteria* were predominant (Fig. 1). At the first sampling time 24.3% and 24.4% of the sequences remained unassigned for the non-Bt and Bt-maize, yielding a total of 2334 and 2777 sequences respectively, while at the second sampling time 22.1% and 22.6% of sequences remained unassigned, with 2177 and 2374 total sequences for the non-Bt and Bt-maize respectively.

The relative presence of the three major phyla was similar in the samples from the third year (Fig. 1), where 22.9% of the sequences were unassigned at the first sampling time for the non-Bt maize (2916 total sequences) and 19.2% for the Bt-maize (3344 total sequences); 21.0% of the sequences were unassigned for the non-Bt maize at the final sampling time, and 22.9% for the Bt-maize, with 4236 and 4523 total sequences respectively.

In the fourth year, an equivalent presence of the predominant phyla was once again found at the first and final sampling time in the non-Bt or Bt-maize, where the relative abundance of *Proteobacteria*, *Actinobacteria* and *Acidobacteria* remained comparatively similar in all cases (Fig. 1). The total number of sequences was 13332 for the non-Bt maize and 24084 for the Bt-maize at the first sampling time, with 15.8% and 16.5% of unassigned sequences respectively. The unassigned sequences were 16.2% for the non-Bt maize and 13.9% for Bt-maize at the final sampling time, with 36314 and 27202 total sequences for the non-Bt and Bt-maize respectively.

**Figure 2:**
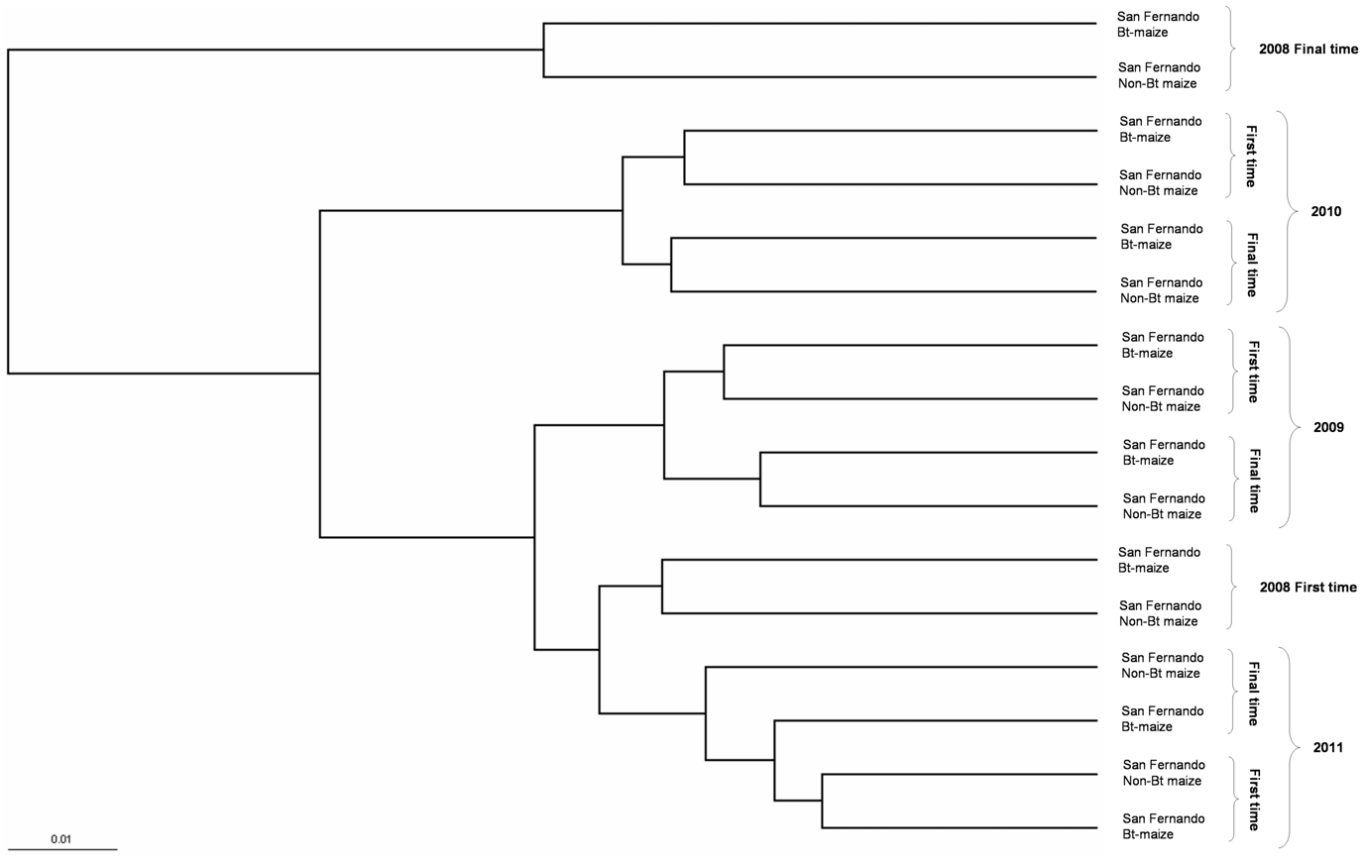
Fast UniFrac hierarchical clustering tree. Analysis of the different soil samples was carried out using normalised abundance weights.

The hierarchical clustering tree of samples based on the UniFrac metric (Fig. 2) shows that the rhizobacterial communities from each particular year are grouped in a separate branch of the tree, except for the year 2008, where samples from the first sampling time were grouped with samples from 2011, and samples from the final sampling time appeared as an independent branch of the tree, as they differ in taxonomic composition (Fig. 1). Samples are also grouped within each year depending on the sampling time. The UniFrac significance test showed no statistical differences between the non-Bt and Bt-maize within each year at any sampling time.

When estimating bacterial diversity, we have previously shown that ESPRIT as well as the web-based workflow, RDP pyrosequencing pipeline, produced the more accurate equivalent results as to the size and length of the hypervariable V6 region of the 16S rDNA sequences obtained from the non-Bt and Bt-maize rhizospheres when compared to other methodologies of analysis [20], therefore, we have used both of them in a comparative manner. Table 1 shows a comparison of the OTUs analyses performed using the RDP pyrosequencing pipeline compared to using the ESPRIT package, at three different dissimilarity levels. Species richness was determined using the Chao1 and ACE estimators for the ESPRIT software, and only Chao1 for the RDP software, since it does not use the ACE estimator (Table 1). No significant differences were detected among the bacterial communities from the non-Bt or Bt-maize, or from one sampling time to another or even from one year to another. The number of OTUs estimated when using the ESPRIT package was always slightly lower, as previously reported [19], however the Chao1 estimator was slightly higher when calculated with RDP, probably due to its own implementation of the algorithm. In all cases the results were consistent irrespective of the methodology used.

**Table 1:** Similarity-based OTUs and species richness estimates at 3%, 5% and 10% dissimilarity levels.

					OTUs	ACE	Chao1						OTUs	ACE	Chao1
**2008**	**First time**	**non-Bt**	**RDP**	**0.03**	1148	NC	2480±279	**2010**	**First time**	**non-Bt**	**RDP**	**0.03**	952	NC	2126±266
				**0.05**	846	NC	1608±164					**0.05**	765	NC	1429±172
				**0.1**	527	NC	670±49					**0.1**	465	NC	668±77
			**ESPRIT**	**0.03**	1031	1842.63	1895±194				**ESPRIT**	**0.03**	916	2117.53	1894±227
				**0.05**	728	1110.54	1065±98					**0.05**	745	1531.48	1430±182
				**0.1**	316	341.97	333±14					**0.1**	433	568.97	534±44
		**Bt**	**RDP**	**0.03**	1135	NC	2469±272			**Bt**	**RDP**	**0.03**	920	NC	2199±297
				**0.05**	918	NC	1528±173					**0.05**	711	NC	1289±156
				**0.1**	541	NC	745±81					**0.1**	425	NC	592±69
			**ESPRIT**	**0.03**	1035	2075	2009±215				**ESPRIT**	**0.03**	886	2228.54	1970±259
				**0.05**	795	1309.08	1269±126					**0.05**	692	1315.88	1179±136
				**0.1**	447	549.7	540±42					**0.1**	406	543.15	529±54
	**Final time**	**non-Bt**	**RDP**	**0.03**	1053	NC	2820±406		**Final time**	**non-Bt**	**RDP**	**0.03**	1011	NC	2414±311
				**0.05**	797	NC	1687±211					**0.05**	815	NC	1595±196
				**0.1**	498	NC	703±50					**0.1**	537	NC	807±94
			**ESPRIT**	**0.03**	982	1951.57	2087±260				**ESPRIT**	**0.03**	1010	2508.72	2201±260
				**0.05**	755	1247.91	1303±153					**0.05**	805	1556.53	1384±146
				**0.1**	415	502.03	506±44					**0.1**	469	642.18	630±65
		**Bt**	**RDP**	**0.03**	1216	NC	2230±258			**Bt**	**RDP**	**0.03**	999	NC	2420±317
				**0.05**	963	NC	1474±179					**0.05**	819	NC	1783±243
				**0.1**	568	NC	679±71					**0.1**	493	NC	688±73
			**ESPRIT**	**0.03**	1029	1992.92	1947±204				**ESPRIT**	**0.03**	1015	2679.5	2341±293
				**0.05**	726	1095.15	1013±84					**0.05**	821	1692.52	1596±196
				**0.1**	326	352.58	342±14					**0.1**	485	632.34	609±51
**2009**	**First time**	**non-Bt**	**RDP**	**0.03**	1307	NC	2565±350	**2011**	**First time**	**non-Bt**	**RDP**	**0.03**	898	NC	2138±296
				**0.05**	1033	NC	1661±204					**0.05**	691	NC	1300±171
				**0.1**	608	NC	663±85					**0.1**	429	NC	606±71
			**ESPRIT**	**0.03**	1105	2395.43	2287±249				**ESPRIT**	**0.03**	847	1916.25	1774±228
				**0.05**	865	1583.68	1602±186					**0.05**	680	1265.76	1204±149
				**0.1**	473	602.51	593±51					**0.1**	398	523.2	517±53
		**Bt**	**RDP**	**0.03**	1184	NC	2454±253			**Bt**	**RDP**	**0.03**	884	NC	2253±332
				**0.05**	910	NC	1650±178					**0.05**	690	NC	1430±208
				**0.1**	503	NC	654±60					**0.1**	407	NC	540±58
			**ESPRIT**	**0.03**	1104	2315.27	2303±255				**ESPRIT**	**0.03**	852	1990.86	2068±609
				**0.05**	847	1445.27	1448±154					**0.05**	651	1178.58	1194±160
				**0.1**	470	574.01	556±39					**0.1**	360	441.06	429±35
	**Final time**	**non-Bt**	**RDP**	**0.03**	1252	NC	3070±395		**Final time**	**non-Bt**	**RDP**	**0.03**	902	NC	2068±273
				**0.05**	1010	NC	1897±241					**0.05**	717	NC	1318±161
				**0.1**	596	NC	819±76					**0.1**	411	NC	512±53
			**ESPRIT**	**0.03**	1063	2108.05	1899±179				**ESPRIT**	**0.03**	853	1961.8	1730±213
				**0.05**	770	1266.42	1234±128					**0.05**	675	1235.8	1095±119
				**0.1**	362	397.08	387±18					**0.1**	370	460.93	433±32
		**Bt**	**RDP**	**0.03**	1204	NC	2730±301			**Bt**	**RDP**	**0.03**	868	NC	2184±326
				**0.05**	904	NC	1636±176					**0.05**	665	NC	1208±155
				**0.1**	539	NC	728±71					**0.1**	380	NC	529±65
			**ESPRIT**	**0.03**	996	1808.36	1810±186				**ESPRIT**	**0.03**	828	1925.67	1793±242
				**0.05**	716	1112.28	1062±101					**0.05**	648	1211	1153±149
				**0.1**	338	367.01	354±13					**0.1**	357	456.86	444±43

The species richness estimates were determined using the RDP pyrosequencing pipeline or the ESPRIT program, as described in Materials and Methods. NC  =  non computable.

## Discussion

In this study the potentially cumulative effect of Bt-maize cultivation on rhizobacterial communities has been monitored over a four-year continuous cultivation of MON810 maize. The effect of Bt-maize is not only exerted by the exudation of the Cry1Ab toxin through the roots [4], as it is constitutively produced within the whole plant and Cry proteins may also confer unexpected properties to the plant, for example, by increasing lignin content [21] or provoking gene instability at or around the insertion site of the Bt coding gene in the plant genome [22]. Furthermore, transgene rearrangements may occur with a potential to produce changes in plant gene expression and phenotype [23]. The fate and effects of insect-resistant toxin in soil ecosystems have been reviewed [2], and the Cry1Ab protein (event MON810) has been detected to remain in soils after four years of Bt-maize cultivation in field conditions, whereas other Cry proteins as Cry3Bb1 (event MON863) were not [24]. Cry proteins are rapidly adsorbed to clay minerals, which render the proteins resistant to biodegradation in soil, thus facilitating a potential longer exposure of non-target organisms to the toxin [2], [25], [26]. The effect of Cry root exudates on different soil non-target organisms (namely earthworms, nematodes and protozoa) has been investigated extensively with apparently little significance or none all, while infective fungal mycorrhizae could colonize Bt maize roots more efficiently than non-Bt ones, and so the persistence of Cry proteins in soil may also be related to the decrease of some microbial activity [2].

There has been an increasing concern in recent years with respect to altering the composition of soil microbial communities and the studies performed differ in their objective and methodology. This four-year study was conducted to get a further insight into the possible harmful effect that the continued presence of Bt-toxin may exert on the structure of maize rhizobacterial communities.

In our study we found three predominant phyla in all the rhizospheres: *Proteobacteria*, *Acidobacteria* and *Actinobacteria*. The *Proteobacteria* are of great importance to global carbon, nitrogen and sulfur cycling [27]; *Actinobacteria* are an important component of soil communities playing a major role in organic matter turnover in soils, due to their ability to decompose organic materials [28]; *Acidobacteria* are commonly detected in soils [29] and it has been suggested that members of this phylum are likely to play a relevant role in conducting processes in terrestrial ecosystems [30].

The overall distribution of these three phyla did not change from the non-Bt to the Bt-maize at any sampling time. The reason why the structure of the rhizobacterial community changed at the final sampling time in the first year is still unknown, however, a particular climate change comprising a heavier period of rainfall between the first and final sampling times was registered, when almost three times more rain accumulated than during the same period in subsequent years (http://clima.meteored.com). At this particular sampling time the *Actinobacteria* became very predominant, which is compensated mostly by a reduction in *Proteobacteria*, and to a lesser extent in *Acidobacteria* (Fig. 1). It is not infrequent to see that climate changes alter the structure of rhizobacterial communities, and this has also been reported in maize long-term cultivation studies [31]. It is also remarkable to observe the natural resilience of the rhizobacterial communities, which at the beginning of the second year had recovered completely from the climatic episode.

No consistent statistically significant differences have been reported in the number of different groups of microorganisms, enzyme activities or pH between non-Bt and Bt-maize rhizospheres [2], or in soil improved with Bt-maize biomass [32]. Other short-term experiments using techniques such as Phospholipid Fatty Acid profiles (PLFA) [33], polymerase chain reaction–denaturing gradient gel electrophoresis (PCR–DGGE) [34], automated ribosomal intergenic spacer analysis (ARISA) [35] or genome-wide commercially available DNA microarrays [36] concluded that the effects of transgenic Bt-maize on the bacterial community structure are minimal, and that the growth stage of plant or environmental factors may exert a more noticeable effect on the microbial community. The UniFrac statistical phylogenetic analysis did not show significant differences between the non-Bt maize and Bt- maize rhizospheres at any sampling time, and grouped the samples according to the sampling time, confirming the trends observed in the taxonomic analysis (Fig. 2).

The bacterial diversity analysis showed no differences in species abundance or richness between non-Bt and Bt-maize at any sampling point estimated with the RDP pyrosequencing pipeline or the ESPRIT software (Table 1). Moreover, no differences were found throughout the four-year experiment despite changes in the community structure. Although the two methods used to analyse bacterial diversity showed some minor differences, both of them offered consistent results when estimating the diversity of the bacterial communities. These two methods produced qualitatively equivalent results and have been shown to be an adequate choice, both being equally suitable for assessing the diversity of rhizobacterial communities.

Some studies have suggested that the repeated cultivation of transgenic Bt-plants may lead to the accumulation and persistence of Bt proteins in soil [26], [32], [37]. Taking together the results obtained we can conclude that no effects may be attributed to the transgenic Bt-maize when compared to its respective isogenic counterpart over a four-year period of seasonal cultivation. The differences perceived in the composition of the rhizobacterial communities were most likely due to the fluctuations in climate, which affected the non-Bt and Bt-maize almost equally. Nevertheless, further studies concerning soil microbial communities functioning should contribute to a better understanding of the relationship of the bacterial communities with the plant throughout its development.

## Materials and Methods

### Plant Materials and Sampling

Bt-maize MON810, variety DKC6451YG, expressing the Cry1Ab protein (Monsanto Agricultura, Spain) and its isogenic non-Bt line DKC6450 (Monsanto Agricultura, Spain) were grown in experimental maize fields located in San Fernando de Henares, Madrid, Spain (N40° 25′ 14″ W3° 29′ 30″). Current agricultural practises were maintained throughout the four years of cultivation period and crop residues were removed after each vegetative cycle. The surface of each experimental field was 40 m^2^; both fields were annexed to each other and separated by a four meters wide path. Four extra rows of non-Bt maize surrounded both fields. The non-Bt and Bt-maize plants were harvested at two different growth stages: about 90 days after seeding (first sampling time), when the plants had around 8 leaves, and just before crop harvesting at final growth (final sampling time). The four extra rows were not sampled. Roots and adhered soil measuring approximately 2 mm or less in diameter were separated from the bulk soil by gently shaking the root system. The term “rhizosphere” describes the carefully separated soil adhered to these roots. Given the small size of the maize fields, these were divided into subplots and 3 samples were taken from each subplot at the time of collection. A total of 9 subplots were collected from each maize field at every collection and an equal amount of soil from each of the 27 samples was pooled in all cases. The experiments were performed during development throughout 2008, 2009, 2010 and 2011. The performed studies did not involved human or animal participants and, in this regard, did not required specific permits.

**Table 2:** Multiplex identifiers (MIDs).

Year	Samplig time	MID Bt-maize	MID Non Bt-maize
2008	First	MID1 (5′-ACGAGTGCGT-3′)	MID2 (5′- ACGCTCGACA -3′)
	Final	MID3 (5′- AGACGCACTC -3′)	MID4 (5′- AGCACTGTAG -3′)
2009	First	MID5 (5′-ATCAGACACG-3′)	MID6 (5′- CGTGTCTCTA -3′)
	Final	MID7 (5′- CGTGTCTCTA -3′)	MID8 (5′- CTCGCGTGTC -3′)
2010	First	MID1 (5′-ACGAGTGCGT-3′)	MID2 (5′- ACGCTCGACA -3′)
	Final	MID3 (5′- AGACGCACTC -3′)	MID4 (5′- AGCACTGTAG -3′)
2011	First	MID1 (5′-ACGAGTGCGT-3′)	MID2 (5′- ACGCTCGACA -3′)
	Final	MID3 (5′- AGACGCACTC -3′)	MID4 (5′- AGCACTGTAG -3′)

The Multiplex identifiers (MIDs) used for pyrosequencing the different samples are shown.

### Texture and Chemical Properties of the Soils

The texture of the soil appears to be loamy containing 16.5% clay, 50% silt and 33.5% sand, as determined by Agriquem S.L. (Seville, Spain). Average organic matter (OM) content was 3.03% with a Cationic Exchange Capacity of 4.7 meq/100 g. Total rainfall registered in the experimental field over the four-year study was collected from http://clima.meteored.com.

### DNA Extraction, PCR Amplification and Pyrosequencing

Rhizospheres from each of the different collection times were pooled and the soil was subjected to three independent DNA extractions using the PowerMax Soil DNA kits (MO Bio Laboratories Inc., USA) following instructions from the supplier. Soil DNA from each of the three independent extractions was used as template for PCR amplification of the V6 hypervariable region of the 16S rRNA gene. The oligonucleotide design included 454 Life Science’s Titanium A or B sequencing adaptors fused to the 5′ end of primer 967F (5′- CAACGCGAAGAACCTTACC –3′) and 1046R (5′- CGACAGCCATGCANCACCT –3′), where a MID (Multiplex Identifier) was included immediately preceding the V6 specific primer, allowing the samples to be analysed in a single lane of the 454 pyrosequencer first sampling time. A different MID was included for each sample as indicated in Table 2.

PCR amplification was performed by incubation at 95°C for 5 min, followed by 30 cycles of incubation at 95°C (30 sec), 63°C (45 sec) and 72°C (1 min), with a final extension cycle of 5 min at 72°C. The amplified DNA resulting from the three independent PCR reactions for each DNA template preparation was pooled and cleaned (Illustra GFX PCR DNA purification kit, GE Healthcare), checked with Bioanalyser 2100 (Agilent technologies), quantified with Quant-IT-picogreen (Invitrogen) and used to make the single strands on beads, as required for 454 Titanium pyrosequencing [38]. The obtained sequences were deposited in the NCBI sequence reads archives (accession number SRA009281).

For taxonomical purposes, the 454 reads for the V6 regions of each soil were filtered to eliminate the short sequences that account for 50% of all pyrosequencing errors [17] and then compared with the RDP database version 10 [12] using BLASTN. Files containing the 25 best matches for each of the 454 determined sequences were used as input to generate the corresponding taxonomic trees by means of the MEGAN 2.0 program [13].

Fast UniFrac (http://bmf2.colorado.edu/fastunifrac) [14], [15], [16] was used to perform a hierarchical clustering of the samples based on their phylogenetic distances. The analysis was conducted with the Greengenes core as a reference phylogenetic tree, Jackknife supporting values, and calculated using normalized abundance weights. The Fast UniFrac significance test was used to assess the existence of statistically significant differences among the 16S rDNA sequences from each soil sample, based on their phylogenetic distances.

To assess taxonomic-independent diversity, an equal number of sequences (1959) were randomly selected from each soil, and the selected pools of sequences were procesed with the RDP pyrosequencing pipelines (http://pyro.cme.msu.edu), which build an initial MSA using the Infernal tool [39] and then proceed directly to perform a complete linkage clustering to cluster OTUs (operational taxonomic units), and calculate species richness with the Chao1 estimator. The obtained results were compared with those generated by the ESPRIT software (using the *–*f parameter), which avoids the initial multiple sequence alignment step by applying an efficient k-tuple based distance filter, subsequently aligning the sequences using the Needleman-Wunsch method and computing pairwise distances using the quickdist algorithm [19]. Hence, a comparison is made of the efficiency of both methods and species diversity estimation. Regarding species richness estimation, confidence intervals were calculated at a 95% confidence level for all Chao1 data.

## Supporting Information

Figure S1 **Taxonomic trees.** Taxonomic trees resulting from pyrosequencing the V6 region of the 16S rDNA extracted from each field at the indicated sampling times are shown. The size of the dots reflects the relative amount of taxa assigned to each particular node.
(TIF)
